# Effectiveness of integrated tuberculosis and depression treatment led by non-specialist health workers versus standard care among persons with tuberculosis in a low-resource setting: A quasi-experimental non-randomized controlled trial

**DOI:** 10.1371/journal.pmen.0000636

**Published:** 2026-09-21

**Authors:** Ngozi Murphy-Okpala, Charles Nwafor, Chinwe Eze, Martin Njoku, Okechukwu Ezeakile, Anthony Meka, Ngozi Ekeke, Francis Iyama, Daniel Egbule, Chukwuma Anyaike, Obioma Chijioke-Akaniro, Joseph Chukwu, Beatrice Kirubi, Jacob Creswell, Justus Uchenna Onu

**Affiliations:** 1 RedAid Nigeria, Enugu, Enugu, Nigeria; 2 Global Health Department, DAHW – German Leprosy and Tuberculosis Relief Association, Abuja, Nigeria; 3 Department of Public Health, Federal Ministry of Health& Social Welfare, Abuja, Nigeria; 4 National Tuberculosis, Leprosy and Buruli Ulcer Control Program, Federal Ministry of Health & Social Welfare, Abuja, Nigeria; 5 Stop TB Partnership, Geneva, Switzerland; 6 Department of Mental Health, Nnamdi Azikiwe University, Awka, Anambra, Nigeria; University of Canterbury, NEW ZEALAND

## Abstract

This study determined the effectiveness of integrated TB and depression care compared to standard TB care in reducing depressive symptoms over time in persons treated for TB. This was a quasi-experimental, non-randomized controlled trial involving all TB cases with significant depressive symptoms (PHQ score ≥ 5) in ten already existing sites (i.e., eight intervention sites and two control sites) in Nigeria. At the intervention sites, non-specialist health workers (NSHWs) delivered treatment for both TB and depression while the NSHWs in the control sites delivered TB treatment and then referred patients with depression to a specialist in a different setting for treatment. Changes in depression symptom scores were measured at baseline, 2^nd^, 8^th^ and 24^th^ weeks using Patient Health Questionnaire (PHQ-9). Mixed-ANOVA design was used to determine changes in PHQ-9 scores between-subjects and within-subjects across intervals of follow-up using both the per protocol and intent-to-treat analyses while controlling for covariates such as age, social support, sex, education. There were no significant differences in the PHQ-scores between the two groups (F = 0.95, p = 0.32). In addition, across the intervals of follow-up, PHQ-scores (i.e., depressive symptom scores) decreased significantly with time in both the intervention and control arms (p < 0.001). Furthermore, the interaction between the study arms and time was not significant (F = 0.66, p = 0.57), indicating that the groups were changing over time in a similar way. Participants with marked decrease in depressive symptoms completed their TB treatment and achieved cure when compared to the higher scores seen in those who dropped out or died or were transferred (F = 25.78, p < 0.001). The findings indicated that TB and depression treatment delivered by NSHWs significantly reduced depressive symptoms across intervals of follow-up. In addition, relief from depressive symptoms was significantly associated with completing TB treatment and achieving cure. Clinical Trial Number: PACTR202501828312848.

## Introduction

Tuberculosis (TB) is a leading cause of morbidity and mortality worldwide [1]. In 2024, an estimated 1.23 million persons died from tuberculosis, making it the world’s leading cause of death from a single infectious agent and among the top leading causes of death globally [1]. This dreary picture of TB is made worse by its comorbidity with depression; a commonly occurring mental health condition expected to be the most burdensome disorder by 2030 [2,3].

Depressive disorders is highly prevalent among individuals diagnosed with tuberculosis, with empirical estimates spanning from 11.3% to 80.2% [4,5]. This substantial variation across epidemiological data is likely driven by methodological heterogeneity across studies, particularly regarding case ascertainment strategies (e.g., diagnostic versus screening tools), and other sample characteristics such as the stage in TB treatment and presence of comorbidities. In Africa, a recent meta-analysis reported that about four out of every ten persons with TB have significant depressive symptoms [5]. Depression has been identified as a possible contributor to TB and multi-drug resistant TB (MDR-TB) epidemics [2]. This observation is predicated on the finding that depressed individuals with TB are less likely to seek care promptly, if at all, and if treatment is sought, they are less likely to adhere to treatment requirements [2]. This behavior has implications for TB control programs in sub-Saharan Africa, leading to drug resistance, morbidity and mortality and continued community transmission [2].

Despite the known mental health burden in this population, integrated mental health services are largely unavailable to persons with TB in Nigeria. Unfortunately, treatment gaps for mental health conditions in Nigeria is already high in the general population, almost approaching 80% [6], and is expected to be even more in disadvantaged populations such as those with TB. These unmet mental health needs are driven in part by limited access to care and providers’ shortages [6]. One significant barrier to accessing mental health care is the inadequate number of mental health specialists with less than 200 psychiatrists for a population of >200 million [7]. Most psychiatric services in Nigeria are hospital-based, in academic tertiary centers mostly in big cities, even though more than 50% of Nigerians live in rural and sub-urban areas where access to any specialized mental health provider is almost non-existent [7].

Although evidence-based interventions for depression exist, integrating and sustaining them in TB care remains a major challenge due to the shortage of trained providers and the fragmentation of health services [7,8]. Task sharing, defined as – a rational redistribution of tasks has been shown to improve access to health care especially in low-resource settings and underserved populations [9]. There is evidence of effectiveness of integrated mental interventions delivered by non-specialist health care workers in both communicable and non-communicable disease settings (e.g., HIV/AIDS) and cancer), respectively [10]. In addition, there is some evidence of the non-inferiority of non-specialist mental health delivered interventions when compared with specialist-led interventions [11]. However, the evidence is sparse in TB population, hence, the need for more studies in this area.

More so, whether an integrated TB plus depression treatment led by non-specialist health workers (NSHWs) is more effective in reducing depressive symptoms when compared to standard care for TB and depression deserves investigation in our setting. This is particularly important because of the several gains in task-sharing such as expanding mental health service access, reducing treatment gaps, lowering stigma, improving outcomes for both conditions and cost-effectiveness [9]. Based on the foregoing, we determined the effectiveness of integrated TB and depression treatment compared to standard TB care in reducing depressive symptoms over time in persons treated TB.

## Methodology

### Ethics statement

Ethical approval was obtained from the Health Research and Ethics Committee of the University of Nigeria Teaching Hospital, Enugu, Nigeria, with reference number UNTH/HREC/2024/12/4003. International ethical norms and standards were strictly adhered to; written informed consent was obtained from all participants. Participants were informed that they were free to withdraw from the study at any time, even after having consented initially, and this, did not in any way affect the person’s medical care. Information gathered in the course of the research were stored in an encrypted computer accessible only to the researchers.

**Study design**: This study was part of a project to integrate mental health services into tuberculosis care, the study protocol has been published elsewhere [12]. We utilized a quasi-experimental, non-randomized controlled trial to evaluate the effectiveness of an integrated one-stop treatment for TB and depression by NSHWs in reducing depressive symptoms when compared to standard TB care. The choice of a quasi-experimental design was based on the anticipated small number of participants and the constraints posed by program timeline, making a randomized controlled trial (RCT) not very appropriate for this phase. However, the study protocol was registered with the Pan African Clinical Trials Registry with trial number of PACTR202501828312848 available at https://pactr.samrc.ac.za/Search_v2.aspx.

**Setting**: The study was implemented within the TB REACH wave 10 (TBRW10) project carried out in three states in Nigeria (Anambra, and Enugu in southern Nigeria, and Nasarawa in Northern Nigeria). The study involved ten Local Government Areas (LGAs) in Nigeria (i.e., eight in the South and two in the North). By the project design, the Southern sites (i.e., 11 facilities across eight LGAs) were the intervention arm while the Northern sites (i.e., seven facilities in two LGAs) were the control arm. In the TBRW10 project, screening officers (i.e., nurses, community health extension workers and directly observed therapy counsellors) in selected (public and private) primary, secondary and tertiary health facilities were trained to conduct integrated screening for both TB and common mental disorders such as depression. The use of both public and private facilities were to ensure the diversity of the population to enhance representativeness. The diagnosis of TB in both the intervention and control arms follow the standard WHO guidelines as adapted in the national TB guidelines. TB cases notified may be bacteriologically confirmed, i.e., those having at least one sputum test with a positive acid-fast bacillus (AFB) and/or positive Mycobacterium Tuberculosis (MTB) and Rifampicin (RIF) resistance (Xpert MTB/RIF) test. They could also be clinically diagnosed TB cases, defined as having a negative sputum AFB or Xpert MTB/RIF test in the presence of compatible clinical symptoms and suggestive chest radiographic findings, as determined by the facility physician. The control LGAs were judged suitable based on similarity in demographic and epidemiological characteristics with the intervention sites. Notably, geographical locations of the control LGAs were not contiguous with any of the intervention LGAs. The geographical distance between two arms reduced the possibility of contamination. The choice of the LGAs reflect a purposeful selection strategy based on population size, TB burden, demographic similarity (i.e., rural and semi-urban mix), geographic separation, and health-system characteristics (i.e., primary health care-level facilities). The study prioritizes comparability of populations and epidemiological context rather than equal numbers of local governments, making the control group appropriate for assessing the intervention impact.

### Interventions

**Intervention Arm (Tuberculosis treatment plus depression onsite):** This arm received integrated TB care (i.e., screening and treatment for both tuberculosis and depression delivered by NSHWs in a one-stop shop TB facility). The psychosocial interventions given by the NSHWs (i.e., community health workers and non-mental health nurses) were psychoeducation, which covered: (a) the working diagnosis; (b) available treatment and duration; (c) importance of treatment adherence; (d) potential side effects; and (e) overview of the care team. Following the World Health Organization Mental Health-Gap Action Program Intervention Guide (mhGAP-IG) [13], they identified the patient’s psychosocial stressors, defined and reviewed plans to address the stressors, strengthened social support, facilitated problem solving techniques and promoted the patient’s functioning in their daily activities by encouraging the continuation of regular social, occupational, and economic activities, while offering life skills and social skills training. Antidepressant medications available in the primary health care drug essential list in Nigeria and mhGAP-IG were used if necessary, according to the established guideline. In keeping with task shifting protocol, continuous refresher trainings and remote supportive supervision were provided to the NSHWs by a consultant psychiatrist. Participants were seen at baseline, 2^nd^ week, 8^th^ week and 24^th^ week, mostly corresponding with the time for assessment of tuberculosis treatment (8^th^ and 24^th^ weeks).

**Control Arm (Tuberculosis treatment plus referral to specialist for depression treatment):** This arm received only treatment for tuberculosis from the NSHWs with screening and referral for depressive symptomatology to the nearest specialist mental health provider. The referred participants returned to the NSHWs for their TB care and re-assessment of their depressive symptoms. Participants were seen at baseline, 2^nd^ week, 8^th^ week and 24^th^ weeks as done for the intervention arm. Information gotten from the NSHWs indicated that none of the participants completed their referral to the specialist. They however, maintained their appointment for the TB care.

**Sample Size Estimation and Sampling Techniques:** We anticipated a small sample size based on the duration of recruitment. Therefore, a total population sample was utilized. In other words, all participants with diagnosis of tuberculosis seen from January 2025 to April 2025 with Patient Health Questionnaire (PHQ-9) score ≥ 5 were included consecutively in all the sites [14]. The study was as pragmatic as possible; applying minimal exclusion criteria to allow for participant selection that mirrors real-world setting. The exclusion criteria consisted of those with depression with psychotic features and a definite plan to commit suicide (i.e., suicidal plan).

**Fig 1:**
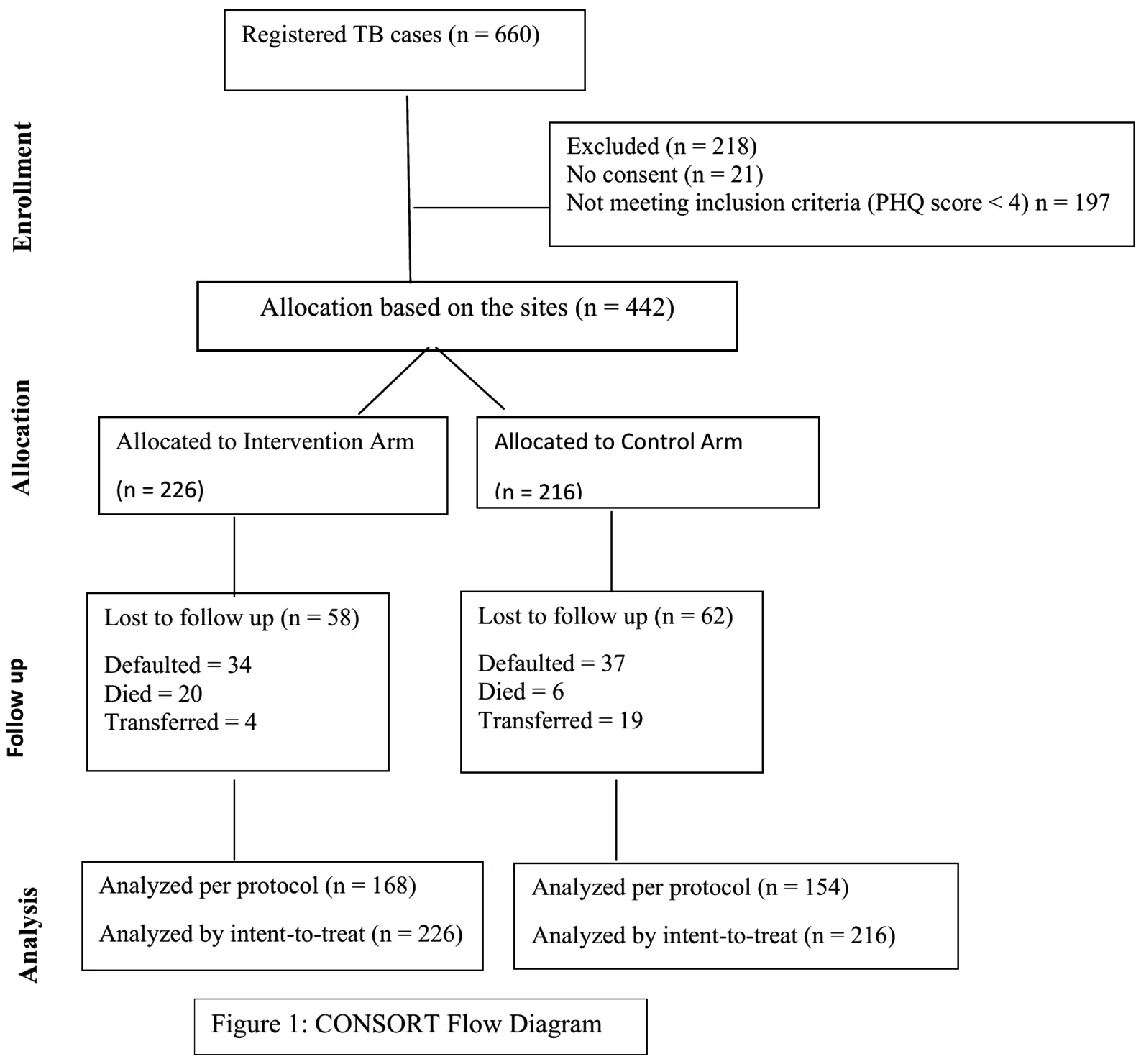
CONSORT flow diagram.

**Procedure:** Participants with TB who had co-morbid significant depressive symptoms in the participating sites constituted the sample population for this study. The program already has ten sites across three states. Eight (8) sites were designated as the intervention arm, where integrated TB and common mental disorders treatment were provided by NSHWs. While the other two (2) sites were designated as the control arm where treatment for TB is going on but patients with comorbid depression were referred out to see mental health specialists. Details of recruitment process are shown in Fig 1.

**Training:** Given that, the present study utilized PHQ-9 and mhGAP-IG, a focused and depression module-specific training was conducted to ensure standardized administration and rating reliability for the 22 NSHWs involved. One of the supervisors (JUO, the last author), who has been trained and has used this tool in previous projects served as the trainer. The NSHWs in the intervention arm were trained in both PHQ-9 administration and using mhGAP-IG to confirm diagnosis and administer both psychosocial and medication therapies. While, the NSHWs in the control sites were only trained on the administration of PHQ-9. However, because of the necessity of the skills acquired from the mhGAP-IG, the control sites were also trained at the completion of the study. The training was structured to emphasize practical skills, interviewer competence, and adherence to standardized diagnostic and treatment procedures. It began with a comprehensive walkthrough of the essential care and practice module of the WHO document, highlighting the fundamentals of good communication. This was followed by a live demonstration by the trainer, who conducted a full-length simulated interview using the PHQ-9 and mhGAP-IG, thereby modeling correct administration and interview dynamics. Subsequently, the trainees engaged in structured role-plays, during which they took turns administering the PHQ-9 and the depression module, playing the role of the respondent, and observing. These interactive sessions provided opportunities for immediate feedback, clarification of errors, and reinforcement of best practices. A series of supervised mock interviews and treatment sessions were conducted, during which each trainee administered the relevant sessions and other study tools to a peer while being observed by the trainer, who provided individualized feedback. The language of training was English, which is the primary language of academic instruction and the language in which the instruments were designed. Nevertheless, attention was paid to ensuring that interviewers are able to appropriately interpret or explain selected terms in locally understood expressions or dialects (e.g., Igbo and Hausa), particularly when interacting with respondents whose comprehension of English is limited. Care was taken to preserve the conceptual integrity of the original questions during such interpretations. The translation was done using a two-stage iterative process. First, a group of three persons with minimum of Junior Secondary Certificate translated from English to the local language (i.e., Igbo or Hausa). Thereafter, the Igbo/Hausa version was back translated to English by another set of three persons who were not part of the first group. In the final phase, the two groups met with the researchers to harmonize their translations.

The training was conducted over a two-day period, comprising approximately 12–14 contact hours in total. The first day was devoted to theoretical orientation, familiarization with the structure of the study tools, live demonstrations, and role-play exercises. The second day was dedicated to supervised mock interviews, review of practical challenges, and standardization procedures. To ensure reliability in the use of the study tools, inter-rater reliability was assessed by having pairs of interviewers independently score the same simulated or recorded interview. Concordance between ratings was examined using percentage agreement or, where applicable, Cohen’s kappa statistic. The main study began when an inter-rater reliability was achieved (Cohen’s Kappa >0.8). In addition, a competency-based checklist was used by the trainer to evaluate each trainee’s performance during mock interviews, focusing on proper navigation of skip patterns, use of standard probes, clarity of questioning, and consistency in coding responses. Before the commencement of actual data collection, a short pilot phase was conducted, involving 3–5 interviews and treatments administered under supervision. This phase provided an opportunity to test interviewer readiness, identify logistical challenges, and make any necessary refinements. Only those research assistants, who demonstrated adequate proficiency, as judged by the trainer, were permitted to participate in data collection. To maintain quality throughout the data collection phase, random interviews were periodically reviewed, to verify adherence to the protocol. Any deviations were addressed through corrective feedback and, where necessary, re-training. This layered approach to training and quality assurance is intended to enhance the reliability, validity, and cultural sensitivity of the data generated through the study tools.

**Questionnaire Administration:** Baseline assessment included rating for depressive symptoms and social support using the PHQ-9 and Oslo social support scale (OSSS), respectively [14,15]. The PHQ-9 was originally designed to improve detection of depression in primary care and non-psychiatric settings [14]. It is scored on a 4-point Likert scale from 0-3 with range of 0–27. Scores ≥ 5 indicate significant depressive symptoms and patients with these scores are enrolled into the study [14]. The OSSS is a brief, self-report questionnaire designed to assess an individual’s level of social support [15]. It typically includes three items that evaluate support from close confidants, sense of concern and practical supports. It is scored on a 5-point Likert scale (1–5) with scores ranging from 3-15. It has been validated for use in Nigeria [16]. Social support, socio-demographic variables (e.g., age, sex, marital status, level of education) and some clinical characteristics (e.g., dyspnea assessed using history) were measured and considered potential covariates.

**Follow-up:** Subsequent follow-up visits were at 2, 8 and 24 weeks. These intervals of follow-up corresponded to the time of expected onset of relief from depressive symptoms (i.e., two weeks), completion of active TB treatment (i.e., eight weeks) and completion of continuation TB treatment (i.e., 24 weeks). Baseline instruments (i.e., PHQ-9 and OSSS) were repeated across intervals of the follow-up. In addition, TB-related outcomes (i.e., completed treatment, interruption, default, and death) was also assessed.

**Data analysis**: Data was analyzed using IBM-SPSS version 20. The normality of distribution of continuous variables was established using an analytical method (e.g., Kolmogorov-Smirnov test) complemented by graphical methods (i.e., histogram and Q-Q plots). Data were analyzed using the per protocol and intent-to-treat methods. The intention-to-treat analysis was used to address attrition or loss to follow-up using the Last Observation Carried Forward (LOCF). The LOCF was done for 120 participants, 58 for the intervention arm and 62 for the control arm. Changes in the depressive symptoms (i.e., as continuous variable) between the two arms across the four (4) intervals of follow-up was compared using repeated measures analysis (i.e., mixed-ANOVA model) while controlling for covariates such as comorbidities, social support and age. Partial eta-squared was used to estimate the effect size of the variance in the dependent variable (i.e., depressive symptoms) explained by the independent variable (i.e., intervention received). Treatment effect was estimated between-subjects using effect size at each point of follow-up. Partial eta-squared score of 0.01 was considered small, 0.06 = medium and 0.14 = large. Multivariable linear regression analysis was used to determine the independent predictors (i.e., age, gender, area of residence, marital status, level of education, and social support) of depression score at 24^th^ week. The association between depression symptom scores and the tuberculosis treatment outcomes were done using Analysis of Variance and Bonferroni Post-Hoc analysis. All tests were two-tailed at 95% confidence interval and p-value was considered significant if less than 0.05.

## Results

Four hundred and forty-two (442) persons participated in the study (i.e., 226 persons in the intervention arm and 216 in the control arm). Males were slightly higher with 239 participants (i.e., about 54%) while females were 203 in number. Other characteristics is detailed in Table 1.

**Table 1:** Socio-demographic characteristics of the participants.

Variables	Control Arm (N = 216) n (%)	Intervention Arm (N = 226) n (%)	Test stat	p-value
**Median age in years (IQR)**	40.00 (21.00)	43.00 (20.00)	U = 21160.00	0.01*
**Sex**			χ^2^ = 0.05	0.81
Male	118 (54.6)	121 (53.5)		
Female	98 (45.4)	105 (46.5)		
**Marital Status**			χ^2^ = 11.47	0.003*
Single	42 (19.4)	57 (25.2)		
Married	166 (76.9)	145 (64.2)		
Separated/Widowed	8 (3.7)	24 (10.6)		
**Area of Residence**			χ^2^ = 93.83	<0.001*
Rural	203 (94.0)	120 (53.1)		
Urban	13 (6.0)	106 (46.9)		
**Level of Education**			χ^2^ = 63.89	<0.001*
No formal	99 (45.8)	32 (14.2)		
Primary	30 (13.9)	37 (16.4)		
Secondary	76 (35.2)	109 (48.2)		
Tertiary	11 (5.1)	48 (21.2)		
**Religion**			χ^2^ = 71.62	<0.001*
Christianity	112 (51.9)	200 (88.5)		
Islam	97 (44.9)	25 (11.1)		
Traditional	7 (3.2)	1 (0.4)		

U = Mann-Whitney U-test, IQR = Interquartile Range, * = Significant p-value.

**Fig 2:**
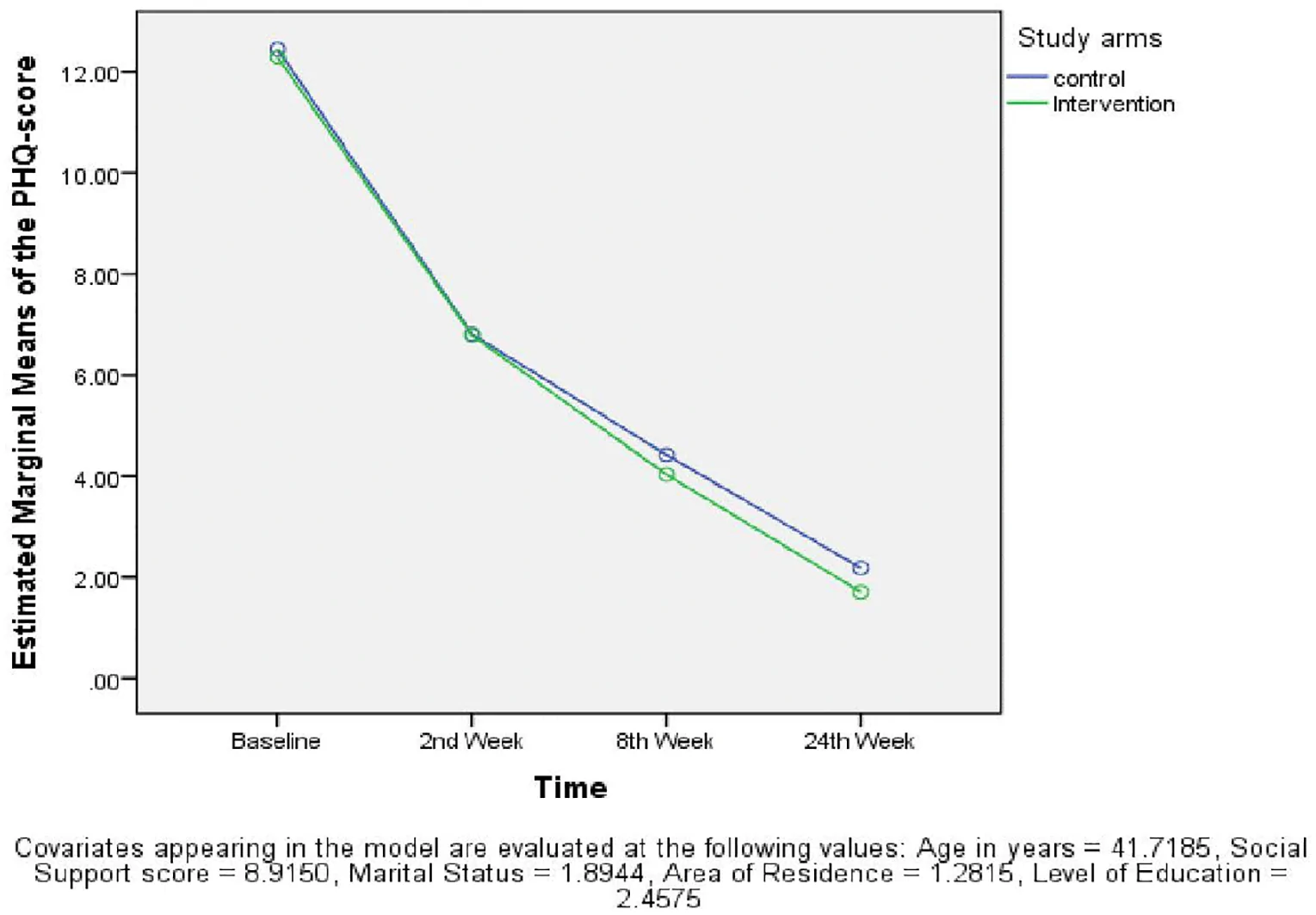
Between-subject and within-subject comparison of depression scores across the intervals of treatment follow-up for per protocol analysis.

**Fig 3:**
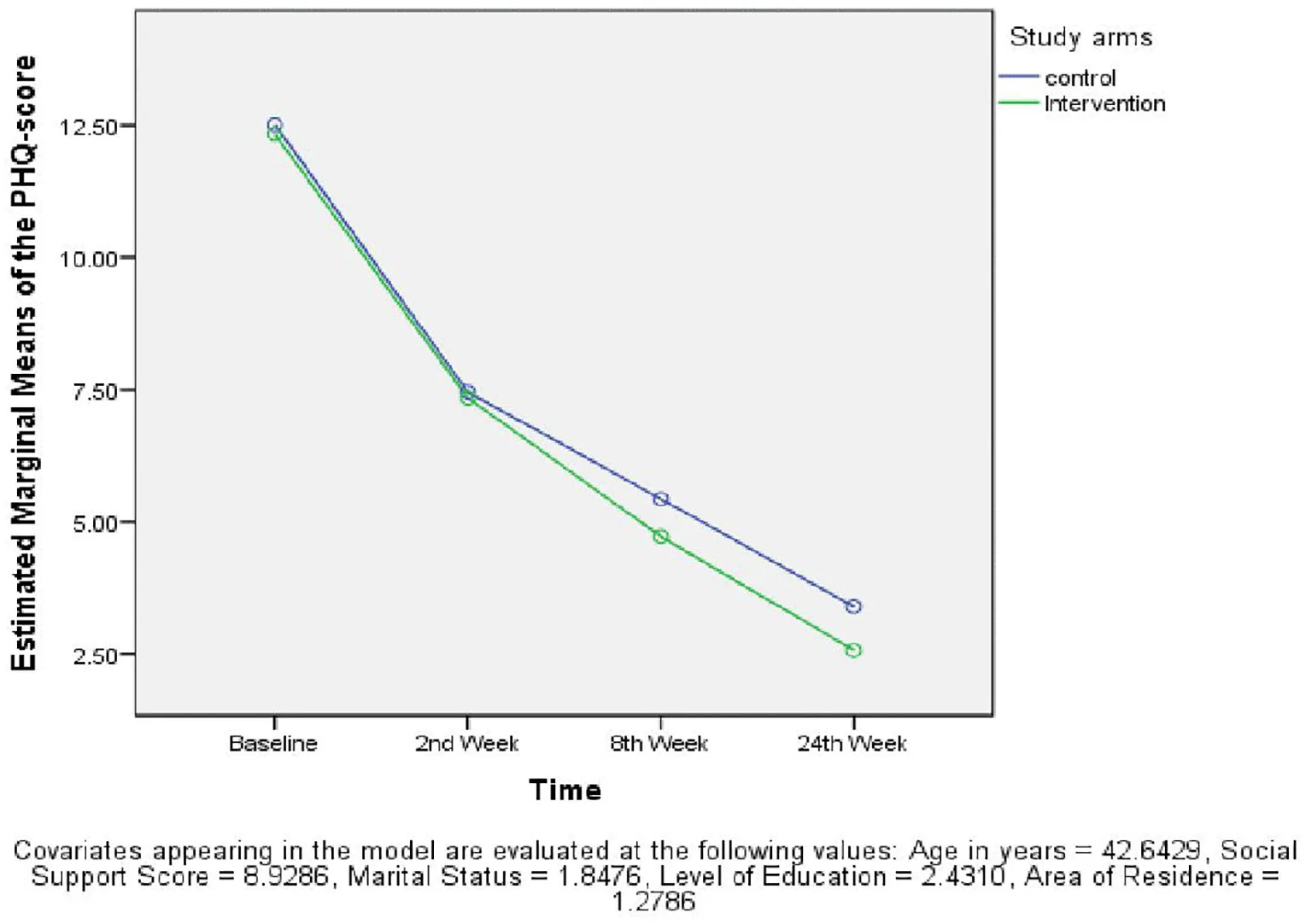
Between-subject and within-subject comparison of depression scores across the intervals of treatment follow-up for intention-to-treat analysis.

Table 1 shows the socio-demographic characteristics of the participants. As shown in the table, the two groups differ significantly in age (p = 0.01), marital status (p = 0.003), area of residence (p < 0.001), level of education (p < 0.001), and religion (p < 0.001). The between-group comparison of PHQ scores at each interval of treatment follow-up is shown in Table 2. The table shows that the PHQ scores between the intervention and control arms did not differ significantly at baseline, 8^th^ and 24^th^ weeks. However, at 2^nd^ week, for both per protocol and intent-to-treat analyses, the control group (i.e., those referred to see the specialist) had significantly lower PHQ score (i.e., less depressive symptoms) when compared to the intervention arm (p = 0.001, effect size = 0.22). Fig 2 and Fig 3 show both the between-group changes (i.e., intervention vs. control) and within-subject changes (i.e., across intervals of follow-up) in the depressive symptoms score while controlling for covariates such as age, sex, marital status, level of education, religion, area of residence and social support. The figures show that there was no significant difference in the PHQ-scores between the two groups (F = 0.95, p = 0.32). However, across the intervals of follow-up (i.e., within-subject comparison), the PHQ-scores (i.e., depressive symptom scores) decreased significantly with time in both the intervention and control arms (p < 0.001). In addition, the interaction between the study arm and time was not significant (F = 0.66, p = 0.57), indicating that the groups were changing over time in a similar way. Table 3 shows that the significant independent predictors of depression symptoms score at the end of follow-up were: the intervention received (β = -0.17, p = 0.004), age (β = 0.18, p = 0.001), rural area of residence (β = -0.16, p = 0.007), having primary education (β = 0.15, p = 0.03), and baseline depressive symptoms score (β = 0.32, p < 0.001). Table 4 shows the association between the depression symptom scores and tuberculosis-related outcomes at termination of follow-up. As shown in the table, the level of depressive symptoms was significantly associated with tuberculosis treatment outcomes (F = 25.78, p < 0.001). This is such that, the depressive symptom scores were lowest among those who completed treatment and achieved cure when compared with those lost to follow-up/died and those transferred out to another TB treatment center (p < 0.001).

**Table 2:** Comparison of the between-subject changes in depressive symptom scores at follow-up intervals.

Time	Study Arm	Per Protocol Analysis Median PHQ-9 Score (IQR)	p-value	Effect size	Intention-to-Treat Analysis* Median PHQ-9 Score (IQR)	p-value	Effect size
**Baseline**	Control Intervention	12.00 (8.00) 12.00 (6.00)	0.52	0.08	12.00 (7.00) 12.00 (7.00)	0.52	0.09
**2^nd^ Week**	Control Intervention	6.00 (9.00) 6.00 (6.00)	0.001	0.22	6.00 (10.00) 7.00 (5.00)	0.008	0.22
**8^th^ Week**	Control Intervention	4.00 (7.00) 4.00 (6.00)	0.60	0.01	4.00 (8.00) 5.00 (5.00)	0.41	0.17
**24^th^ Week**	Control Intervention	0.0(3.00) 0.00 (3.00)	0.68	0.06	0.00 (4.00) 1.00 (4.00)	0.52	0.05

PHQ – Physical Health Questionnaire, IQR = Interquartile Range, * – Last Observation Carried Forward.

**Table 3:** Predictors of depressive symptoms score at the end of the treatment follow-up among the participants.

Dependent Variable	Independent predictors	Standardized β-coefficient	t-test	p-value
**PHQ-9 score at week 24**	**Study Arms**			
	Intervention arm	-0.17	-2.85	0.004*
	Control arm (constant)			
	Age (years)	0.18	3.45	0.001*
	**Sex**			
	Female	-0.07	-1.42	0.15
	Male (constant)			
	**Area of residence**			
	Rural	-0.16	-2.71	0.007*
	Urban (constant)			
	**Education status**			
	No formal	0.15	1.63	0.10
	Primary	0.15	2.12	0.03*
	secondary	0.11	1,44	0.15
	Tertiary (constant)			
	**Religion**			
	Christianity	0.11	0.67	0.50
	Islam	-0.01	-0.10	0.91
	Traditional (constant)			
	**Marital status**			
	Single	0.02	0.38	0.70
	Separated	0.04	0.79	0.42
	Divorced	0.10	1.90	0.05
	Married (constant)			
	Social support	-0.02	-0.59	0.55
	Baseline PHQ-score	0.32	7.22	<0.001*

Coefficient of multiple determination (R^2^) = 0.151, PHQ – Patient Health Questionnaire.

**Table 4:** Comparison of depressive symptom scores with the tuberculosis treatment outcome at the end of follow-up.

Variables	Intention-to-Treat Analysis Mean PHQ-9 score (SD)	Test stat p-value
TB Treatment Outcomes		F = 25.78 p < 0.001**
Completed treatment/cured	1.82 (0.15)	
Loss to follow-up/death	1.64 (0.04)	
Transferred	7.33 (1.11)	

*Post-Hoc pairwise comparison show that the significant difference were between those who completed treatment vs. transferred (p = 0.001) and those lost to follow-up/died vs. transferred (p < 0.001). **Significant difference were completed treatment/cured vs. loss to follow-up/death (p < 0.001), completed treatment/cured vs. transferred (p < 0.001), and loss to follow-up/death vs. transferred (p < 0.04). F = Analysis of Variance, SD = Standard Deviation.

## Discussion

This study utilized a quasi-experimental design to compare integrated TB and depression treatment delivered by NSHWs and standard TB care among patients with TB in Nigeria. The primary aim was to determine the effectiveness of integrated TB plus depression treatment delivered by NSHWs when compared with standard TB care. The strength of the study is the use of pragmatic quasi-experimental design.

The main highlights of the findings are: (1) there was no significant difference in the depressive symptoms score between the intervention and control arms at the end of follow-up; (2) there was significant decrease in the depressive symptoms score with time in both arms; and (3) age, rural area of residence, primary education, receiving any intervention and baseline depressive symptoms score were the significant predictors of depressive symptoms at the end of follow-up.

The finding of no significant difference in depressive symptoms score between the intervention and control arms at the end of follow-up is not surprising. In other words, we found no evidence of a difference in depression treatment delivered by trained NSHWs with those referred to a specialist. Though a direct comparison is difficult as there is paucity of previous studies in TB population that have utilized similar design. However, our results are consistent with previous reports in other populations. For example, Anjara et al [11], reported non-inferiority of non-specialist delivered treatment for depression when compared with specialist-led treatment for mild-to-moderate depressive symptoms in persons with HIV. This has implication for scaling-up mental health services for persons with TB in Nigeria using an integrated approach. This finding, which is consistent with the possibility that NSHWs can effectively deliver treatment for depression to persons with TB, reinforces the task-shifting strategy advocated for optimizing care in this population [9,17]. Interestingly, there are now international and local guidelines for effective use of task shifting and task sharing to scale-up health care services in low-resource settings and underserved populations [17,18]. Nevertheless, it is important to point out that, despite not completing the referral to the specialist for the treatment of their depressive symptoms in the control arm, depressive symptoms improved with TB treatment alone. This finding re-echoes earlier and recent observations that treatment of TB alone may relieve depressive symptoms [19,20]. For example, Rouf and colleagues [18] reported a progressive decline in significant depressive symptoms from 50.5% at baseline through 9.4% at two months to 2.5% at six months of TB treatment alone. This is understandable, considering that participants in this arm had mostly moderate depression (i.e., median PHQ-9 score of 12). Earlier researchers have also reported the antidepressant properties of some medications such as Isoniazid used in the treatment of TB [20].

Another important finding of the index study was the significant decline in depression symptoms score across the intervals of treatment follow-up in the two study arms. This indicates that regardless of the strategy used to deliver the treatment (i.e., either by a non-mental health specialist or standard care), each group experienced a substantial reduction in their depressive symptoms. Previous studies on integrated depression treatment delivered by non-specialist mental health providers in persons with other infectious diseases (e.g., HIV/AIDS) have similar findings [10,21]. These findings offer some optimism and support for integrated mental health and TB treatment in a one-stop shop as currently being piloted in Nigeria.

Concerning the predictors of depression scores among patients with TB at the end of follow-up, we found age, rural area of residence, primary level of education and severity of depression score at baseline as significant predictors. In particular, age positively correlated with PHQ scores at the end of follow-up. This is consistent with available literature that shows that advancing age may be associated with poor response to treatment in persons with depression [22,23]. Area of residence such as living in rural settings and religion are protective of depressive symptoms [24,25].

Furthermore, the persistence of depressive symptoms was significantly associated with negative TB treatment outcomes such as default, death or being transferred for a more specialized TB care. This finding is consistent with the literature and underscores the complex bi-directional interaction between depression and TB [26]. Sweetland and colleagues [2] reported that depression severely worsens TB treatment by causing poor treatment adherence, leading to treatment failures and refractoriness, drug resistance, prolonged infectiousness, and increased morbidity and mortality. The relationship between depression and TB has been described using the terms “syndemic model” and “vicious cycle” where mental and physical illnesses feed off on each creating dangerous outcomes for both conditions. Similarly, Rouf et al [19] reported a small to medium association between depression and treatment failure among persons with TB. This finding has implication for TB control programs. In other words, for success of a TB control program in Nigeria, there is need for a comprehensive integration of active screening and treatment of mental health conditions [2,27–29].

### Limitations

Though the index study utilized quasi-experimental design and pragmatic eligibility criteria, a randomized controlled trial with appropriate allocation concealment is still the gold standard for evaluating the effectiveness of an intervention. In addition, the use of control sites that were socio-culturally different from the intervention may have influenced our findings. Furthermore, though the two arms were comparable based on demographic and epidemiological similarities, the lower number of LGAs in the control arm may raise questions about generalizability. Though the baseline characteristics were adjusted for using statistical means, matching the participants on certain characteristics from the onset may have been more rigorous. Finally, the duration of follow-up of six month, though appropriate for measuring response to treatment, a longer duration would have allowed for determining the sustenance of the interventions benefit.

## Conclusion

The finding of improvement in depressive symptoms among participants in the intervention arm supports the one-stop-shop integrated model currently advocated in Nigeria. The integrated TB plus mental health approach is further reinforced by the finding that the persistence of depressive symptoms was significantly associated with negative TB treatment outcomes. However, our findings also show that treatment of TB alone could relieve depressive symptoms among persons being treated for TB. Future studies may focus on understanding the reasons for the non-completion of referrals to the specialist mental health services in this population and the cost-effectiveness of the integrated care model versus standard care.

## Supporting information

S1 Data Research Data. (SAV)

S1 File CONSORT Checklist. (DOCX)

S1 Text Study Approved Protocol. (DOCX)
