# Supplementary material for: Effectiveness of integrated tuberculosis and depression treatment led by non-specialist health workers versus standard care among persons with tuberculosis in a low-resource setting: A quasi-experimental non-randomized controlled trial
Source: PLOS Ment Health. 2026 Sep 21;3(9):e0000636. doi: 10.1371/journal.pmen.0000636 (PMC13592570; doi:10.1371/journal.pmen.0000636)
Supplement: Supplementary file 2 [file pmen.0000636.s003.docx]

**The Burden of Depression and Stigma among Persons with Tuberculosis within the Integrated Tuberculosis/Mental Health Project – A Multi-site Mixed-Method Design: The Study Protocol**

**Principal Investigator:** Ngozi Murphy-Okpala^a^ (PI)

**Co-Investigators**

Charles Nwafor^a^,Chinwe Eze^a^,Martin Njoku^a^,Okechukwu Ezeakile^a^, Anthony Meka^a^, Ngozi Ekeke^a^,Francis Iyama^a^,Daniel Egbule^a^,Chukwuma Anyaike^b^,Obioma Chijioke-Akaniro^b^, Joseph Chukwu^b^, Beatrice Kirubi^c^, Jacob Creswell^d^, Justus Uchenna Onu^e*^.

**Affiliations:**

**^a^**RedAid Nigeria, German Leprosy and TB Relief Association, Nigeria.

^b^Department of Public Health, Federal Ministry of Health, Abuja, Nigeria.

^c^National Tuberculosis, Leprosy and Buruli Ulcer Control Programme,Abuja, Nigeria

^d^Stop TB Partnership, Nigeria.

^e^Department of Mental Health, Nnamdi Azikiwe University, Awka, Anambra State

**Background**

Tuberculosis (TB) is a leading cause of morbidity and mortality worldwide [1]. In 2022, an estimated 10.6 million persons fell ill with TB, with almost a quarter occurring in Africa with about 26 deaths per 100,000 population in the region [1]. The dreary picture of TB is made worse by its comorbidity with depression and high level of stigma [2, 3].

Depression is a severe mental disorder expected to be the most burdensome by 2030 [4]. Interestingly, there is high comorbidity between TB and depression [5]. In Africa, a recent meta-analysis reported that about four out of every 10 persons with TB have a major depressive disorder [6]. This has implications for the TB control program in sub-Saharan Africa. It has been reported that comorbid depression in persons with TB delays health-seeking behaviour and increases the risk of treatment non-adherence, resulting to high morbidity and mortality rates, drug-resistance and on-going disease transmission [6]. Being female, poor social support, poor educational status, rural residence, high internalized stigma and clinical symptoms such as dyspnoea have been identified as possible risk factors of major depressive disorder in persons with TB [7].

Similarly, TB-related stigma is enormous in the region with almost one out of every two persons with TB reporting perceived stigma in Africa [8]. The high stigmatization of TB is hinged on perceived contagiousness, lack of knowledge about the routes of transmission and transmissibility, and TB/HIV duality [9]. Stigma contributes to psychological morbidity associated with the illness and impedes [public health](https://www.sciencedirect.com/topics/medicine-and-dentistry/public-health) efforts to prevent and manage the disease [10]. The impact of stigma on TB control shares some similarities with that of depressive disorder, namely, diagnostic delay and treatment non-compliance [8]. In other words, each of this condition (i.e., stigma or MDD) independently impact diagnostic and treatment outcomes in persons with TB.

A complex bi-directional relationship exists between TB and depressive disorder [5]. On the one hand TB infection or reactivation and its treatment may precipitate depression, probably through biological (e.g., malnutrition, inflammatory response and dysregulation of the hypothalamo-pituitary adrenal axis, and treatment with drugs such as isoniazid or cycloserine), behavioral (e.g., substance use) and social factors (e.g., stigma, economic adversity and marital issues) [5]. On the other hand, depression may worsen the outcome of TB through various pathways such as increased inflammatory response, immunosuppression, and altered lipid metabolism. In addition, the impact of depression on health-seeking behavior and adherence to medication may limit access and treatment compliance in persons with TB [5]. The above-described interplay between TB and major depressive disorder has implications for treatment. First, it has been hypothesized that adding antidepressant as an adjunct to TB treatment will improve overall outcome [5]. Second, it is also believed that treatment of TB alone in persons with comorbid depression might relieve the depressive symptoms [5]. The latter is the secondary focus of this study, designed as an add-on to an already existing project by RedAid Nigeria, which focused on the integrative care of tuberculosis, depression and substance use disorder in the settings.

RedAid Nigeria is implementing the TBR W10 project using an integrated services model to expand access to person-centered quality care for Tuberculosis (TB), Mental Health (MH), and Substance Abuse Disorders (SD) in ten intervention LGAs across three states in Southeast and North-central Nigeria (Murphy-Okpala et al. unpublished). As evidence from this pilot project is anticipated to influence future adoption or scale-up, it is crucial to assess and document stigma among PWTB and/or MH/SD and other stakeholders. This will enhance understanding of social and contextual factors affecting health-seeking behavior and service delivery among persons with TB. The index protocol is a mini study within the TBR W10 project that will aim to quantify the burden of depression and stigma among persons with tuberculosis.

**Aim:**

The primary aim will be to determine the burden of depression and stigma in persons with TB and explore the understanding of TB-related stigma (at the individual, secondary, and community levels) across various settings. The secondary aim will be to determine the effectiveness of integrated TB plus depression care when compared with TB care as usual on the depressive symptoms across the intervals of treatment follow-up.

**Specific objectives:**

1. To determine the prevalence of major depressive disorder among persons with TB in the study settings.
2. To determine the level and dimensions of stigma at individual, family, community, and healthcare settings towards persons with TB at different stages of care.
3. To understand the phenomenon of TB-stigma across different stakeholders (i.e., patients, family, community and health care workers).
4. To determine the effectiveness of integrated TB plus depression care when compared with TB care as usual on the depressive symptoms across the intervals of treatment follow-up.

**Methods**:

**Study Design and Setting:** We propose a mixed-method design involving quantitative arm and qualitative arm embedded within the on-going TB REACH wave 10 project which is being implemented in 3 states in Nigeria (Anambra, and Enugu in southern Nigeria, and Nasarawa in Northern Nigeria), from July 2023 to August 2024, with a 4-month no-cost extension till December 2024. The study location will be in the TB REACH W10 intervention arm Local Government Areas, which include: Awka North, Awka South, Nnewi North and Onitsha South LGAs in Anambra state and Awgu, Enugu East, Enugu North and Igboeze North LGAs in Enugu state. The control arm include Nasarawa Eggon and Keffi LGAs in Nasarawa state.

In the TBRW10 project intervention area, screening officers in selected (public and private) primary, secondary and tertiary health facilities as well as community health workers (CHWs) were trained to conduct integrated screening for both TB, depression and substance use disorders using TB symptoms checklist, PHQ-2/PHQ-9 and ASSIST tools, respectively.

Presumptive TB cases are guided on sputum collection (or chest x-ray where needed) for GeneXpert MTB/RIF testing for diagnosis, while those diagnosed with depression or substance use disorder co-morbidity are offered psycho-educational counselling. The diagnosis of TB in both the intervention and control areas follows the standard WHO guidelines as adapted in the national TB guidelines. TB cases notified may be bacteriologically confirmed i.e. those having at least one sputum test with a positive acid-fast bacillus (AFB) and/or positive Xpert MTB/RIF test. They could also be clinically diagnosed TB cases, defined as having a negative sputum AFB or Xpert MTB/RIF test in the presence of compatible clinical symptoms and suggestive chest radiographic findings, as determined by the clinic physician. Across the project facilities, an average of 299 TB cases are diagnosed monthly (the project contributes an average of 87 TB cases monthly).

For the control arm, the sites will continue to provide the standard-of-care with separate silo serviceofferings of TB, depression and SD services. While identified presumptive TB are guided on sputum collection (or chest x-ray where needed) for GeneXpert MTB/RIF testing for diagnosis, all patients identified with depressive symptoms and or SD are referred to the nearest facility for mental health interventions.Across the project control facilities, an average of 188 TB cases are diagnosed monthly.

The control LGAs were judged suitable based on similarity in demographic and epidemiological characteristics. Notably, geographical locations of the Control LGAs are not contiguous with any of the intervention LGAs. Both evaluation and control LGAs share similar population characteristics.

**Ethical Consideration**

Ethical approval was obtained from the Health Research and Ethics Committee of the University of Nigeria Teaching Hospital, Enugu, Nigeria, with reference number UNTH/HREC/2024/12/4003. International ethical norms and standards will be strictly adhered to; written informed consent will be obtained from all participants. Participants will be informed that they are free to withdraw from the study at any time, even after having consented initially, and this will not in any way affect the person’s medical care. In addition, they will be informed that if in the process of the research, any previously undiagnosed clinical condition was found, it will be discussed with them and appropriate medical information given including possible referral as appropriate. Information gathered in the course of the research will be stored in an encrypted computer accessible only to the researchers.

**Quantitative Arm**

We have conceptualized this arm into two stages (1 and 2) that are not mutually exclusive.

**Stage 1:**

**Design:** We propose to utilize a cross-sectional design to determine the burden of major depressive disorder, levels and dimensions of stigma and some psychometric properties of stigma scales in our setting.

**Sample size and sampling technique:** The same sample size estimation applies separately to the primary outcomes. The primary outcome is to quantify the burden of depression and stigma among persons with TB. To estimate this with a 95% confidence interval, pooled prevalence of depression in persons with TB of 39.4% [6], 5% error margin and the estimated monthly population of newly-diagnosed TB in the project of 299, we would require a minimum sample of size 165. This sample size will sufficient to evaluate the psychometric properties of the stigma questionnaires. This is because this sample size is above the number required for factor analysis (i.e. between five and seven participants per scale item) [11]. As the Van Rie Self-Stigma scale has the largest number of items (i.e., 12 items), the target sample size of 84 participants (i.e., 7 × 12) will be adequate to evaluate its psychometrics. Although not directly estimating precision and sample size for the secondary outcome (i.e., the effectiveness of one-stop shop integrated TB and depression treatment versus Treatment as usual), we expect to be able to also evaluate them with good precision using all eligible participants per arm. Participants will be recruited consecutively as they present to the study sites if they meet other eligibility criteria. To be eligible to be screened for depression, participants will have a confirmed diagnosis of TB, aged 18 years and above and is a new case. Those with chronic medical conditions (e.g., cancer, HIV) will be excluded. In addition, family members and health care workers involved in the care of persons with TB and some willing community members will be included to address the stigma objective.

**Data Collection:** This will be two-phased, involving all the newly diagnosed patients with TB in the study sites. In phase one, all diagnosed participants will be screened using PHQ-2. A cut-off score of 3 and above will be considered as a positive screen for depression. Participants who screened positive in PHQ-2 will further be interviewed with PHQ-9 to determine if the person meets the criteria for a major depressive disorder. In addition, 20% of those who screened negative to the PHQ-2 will also be evaluated with PHQ-9. Tuberculosis-related self, family (i.e., secondary), community and healthcare workers’ stigma will be assessed using the self, family secondary, community and healthcare workers stigma scales, respectively.

**Study Questionnaires:** PHQ-2 is a 2-item depressive disorder rated in a 4-point Likert scale. It contains the first two items of PHQ-9 with modest validity indices as screener [12]. At a cut-off of 3 points, the sensitivity, specificity and positive predictive validity is 82.9%, 90% and 34.9%, respectively [12]. The PHQ-2 ranges from 0-6. The PHQ-9 is a 9-item multipurpose instrument for screening, diagnosing, monitoring and measuring the severity of depression. The PHQ-9 was originally designed to improve detection of depression in the primary care and non-psychiatric settings [13]. It is scored on a 4-point Likert scale from 0-3 with range of 0-27. Scores 5-9 indicates mild depressive symptoms, 10-14 is moderate, 15-19 is moderately severe, and ≥20 indicates severe depressive symptoms [13]. Studies on PHQ-9 have demonstrated that despite its brevity, it has useful psychometric properties [13, 14]. Diagnostic validity is well established, PHQ scores ≥10 had a sensitivity of 88% and specificity of 88% for major depressive disorder [13]. The stigma scales are TB-specific tools assessing various dimensions of stigma involving self, family, community and healthcare workers [15]. Each is a 5-point scale and have modest psychometric properties [15].

**Questionnaire Translation:** A two-stage translation process will be utilized to translate the PHQ-2, PHQ-9, and self-stigma, family (i.e., secondary), community and healthcare workers stigma scales into Igbo and Hausa languages. First, two bilingual researchers will translate these tools from English to the respective local languages. Secondly, the translated versions will be back-translated into the English Language. Finally, the researchers will meet to harmonize the translations in a consensus meeting. Participants who do not understand English will use the version translated to their local language.

**Data Analysis:** Data will be analyzed using the International Business Machine-Statistical Package for Social Sconces (IBM-SPSS) version 20. Weighted prevalence will be calculated with 95% confidence interval. Severity of depression will categorized using PHQ-9 score as mild (4-9), moderate (10-14), moderately severe (15-19) and severe (20-27) and described using frequency count and percentages. We will describe the burden of self, family, community and healthcare workers’ stigma using previously cut-off scores. Summary statistics of median and interquartile range will be used to describe the scores per item. Exploratory factor analysis will be used to describe the dimensions of stigma in Nigerian setting. While internal consistency of the stigma measures will be evaluated using reliability estimate (Cronbach alpha).

**Stage 2**:

**Design:** We will utilize quasi-experimental design to evaluate the effectiveness of an integrated one-stop treatment of TB plus mental health care on depressive symptoms across the intervals in follow-up in comparison with treatment of TB as usual (i.e., using anti-TB medications and referral to other health facilities for depressive symptoms).

**Procedure:** Participants who will receive the diagnosis of major depressive disorder in the participating sites will constitute the sample population for this stage. The program already has 12 sites across three states. Ten (10) sites is currently designated as the intervention arm where integrated TB and common mental disorders treatment is being piloted. While the other six (2) sites is designated as comparative arm where treatment for TB is going on but mental health comorbidity is usually referred out.

This stage will utilize this existing infrastructure to determine the effectiveness of an integrated TB plus mental health treatment versus treatment as usual. Baseline assessment will include depressive symptomatology and social support using the PHQ-9 and Oslo social support scale (OSSS), respectively. The OSSS is a brief, self-report questionnaire designed to assess an individual’s level of social support. It typically includes three items that evaluate support from close confidants, sense of concern and practical supports. It is scored on a 5-point Likert scale (1-5) with scores ranging from 3-15. Social support, socio-demographic variables (e.g., age, sex, marital status, level of education) and some clinical characteristics (e.g., dyspnea assessed using history) will be measured and considered potential covariates. Subsequent follow-up will be at 2 weeks, 8 weeks and 24 weeks, respectively. These intervals of follow-up correspond to the time of expected onset of relief from depressive symptoms, active and continuation TB treatment. Baseline instruments (i.e., PHQ-9 and OSSS) will be repeated across intervals of the follow-up. In addition, TB-related outcomes (i.e., on treatment, interruption, default and death) will also be assessed. Participants aged 18 years and older, diagnosed with TB and co-morbid depression will be included if they give consent. Patients with suicidal plans or attempts and/or with psychotic symptoms, who plan to transfer their TB treatment elsewhere, which are too ill to participate, who have other co-morbid chronic conditions will be excluded. They will be recruited using consecutive sampling.

**Data Analysis**: Data will be analyzed using IBM-SPSS version 20. Normality of distribution of continuous data will be checked using Shapiro-Wilk test. Data will be analysed using intention-to-treat and par protocol methods. Changes in the depressive symptoms (i.e., as continuous variable) between the two arms across the four (4) intervals of follow-up will be compared using mixed-ANOVA model while controlling covariates such as social support and age. Treatment effect will be estimated between-subjects using effect size at each point of follow-up. Multivariate logistic regression analysis will be used to determine the independent predictors (i.e., age, gender, area of residence, marital status, level of education, and social support) predictors of depression (present/absent) at baseline and six months. All tests will be two-tailed at 95% confidence interval and p-value will be considered significant if less than 0.05.

**Qualitative Arm**

This will utilize exploratory phenomenological approach to understand the phenomenon of stigma in persons with TB. The study will involve in-depth interviews with patients, their family members and key persons within the community. Purposive sampling will be used to select two participants for each category per site, ensuring diversity in terms of age, gender, and other criteria.

**Data Analysis:** The audio recording will be transcribed verbatim and then analyzed thematically according to the recommendation of Braun and Clarke [16]. Transcripts will be carefully read by the lead investigator, then segments of text will be coded, synthesized, and integrated into categories based on similarities of meaning. Codes will be developed through both deductive and inductive processes. Predetermined codes based on the topic list for the interviews and review of the relevant theoretical constructs will form the basis of the development of initial codes (deductive approach). Coding will continue until no new concept is formed. Coding consistency will be checked by one of the co-investigators to ensure trustworthiness, and theme confirmation will be reached after a consensus meeting with other investigators. To be reflexive, authors who are involved in the care of persons with TB will try to be aware and be transparent on how their personal experiences and perspectives may influence the analysis and interpretation of the results. The NVivo software will assist in managing the data. The analysis will focus on identifying key themes related to the dimensions of TB-related stigma and its impact on health-seeking behavior among the participants and their families. Themes will be summarized and interpreted using a contextualist approach where the characteristics of the study setting will be used to understand the experiences of the participant. Quotes will be selected by the principal investigator to exemplify each theme.

Despite the strengths of the index study, there are some limitations. First, the PHQ-9 is considered a screening tool for MDD, using diagnostic tools (e.g., Mini-International Neuropsychiatric Interview or the Composite International Diagnostic Interview) would have been more appropriate for a definitive diagnosis. Though, multiple stakeholders were interviewed for their perspectives on TB stigma, the views of policy-makers, law enforcement and the media were not considered. These perspectives may have allowed for a more robust conversation on the issue of TB-stigma.

**Declarations**

**Ethical Consideration**

Ethical approval was obtained from the Health Research and Ethics Committee of the University of Nigeria Teaching Hospital, Enugu, Nigeria. International ethical norms and standards will be strictly adhered to; written informed consent will be obtained from all participants. Participants will be informed that they are free to withdraw from the study at any time, even after having consented initially, and this will not in any way affect the person’s medical care. In addition, they will be informed that if in the process of the research, any previously undiagnosed clinical condition was found, it will be discussed with them and appropriate medical information given including possible referral as appropriate. Information gathered in the course of the research will be stored in an encrypted computer accessible only to the researchers.

**Funding:**

Funding will be provided by Stop TB Partnership

**Competing interests**

NMO, CN, CE, MN, OE, AM, NE, FI, DE, CA, OCA, JC, BK, and JC get salaries and honariria for TB-based organizations. JUO consults occasionally for RedAid for projects. Otherwise, he has no conflicts of interest.

**Availability of data and materials**

The raw data generated will be made available

**Code availability**

Not applicable

**Authors' contributions:**

NMO, CN, CE, MN, OE, AM, NE, FI, DE, CA, OCA, JC, BK, JC, and JUOwere all involved in the conceptualization of the study. Data collection will be supervised by NMO, CN, CE, MN, OE, AM, NE, FI, and JUO. However, data analysis and interpretation was done by JUO. JUO drafted the initial manuscript; all authors read and approved the final draft of the manuscript.

**Consent to publish**

Not applicable

**Acknowledgements:**

Not applicable

**References**

1. World Health Organization. Global Tuberculosis Report, 2023. Available at <https://www.who.int/teams/global-tuberculosis-programme/tb-reports/global-tuberculosis-report-2023>, accessed on 6^th^ October, 2024.
2. Sweetland A, Oguendo M, Wickramaratne P, Weissman M, Wainberg M. Depression: a silent driver of the global tuberculosis epidemic. World Psychiatry, 2014; 13:325-326.
3. Mbuthia GW, Nyamogoba HDN, Chiang SS, McGarvey ST. Burden of stigma among tuberculosis patients in a pastoralist community in Kenya: A mixed methods study. PLoS One. 2020; 15(10):e0240457. doi: 10.1371/journal.pone.0240457.
4. Funk M. Global burden of mental disorders and the need for a comprehensive, coordinated response from health and social sectors at the country level. Retrieved on October, 2024.
5. Zhang K, Wang X, Tu J, Rong H, Werz O, Chen X. The interplay between depression and tuberculosis. Journal of Leukocyte Biology, 2019; 106 (3):749-757 <https://doi.org/10.1002/JLB.MR0119-023R>.
6. Amha H, Mulugeta H, Getnet A, Haile D, Anteneh K, Tilahun M et al., Magnitude of depression and associated factors among patients with tuberculosis in sub-Saharan: A systematic review and meta-analysis. Journal of Affective Disorders Reports, 2021; 100080 https//doi.org/10.1016/j.jadr.2021.100080.
7. Shen R, Zong K, Liu J, Zhang L. Risk Factors for Depression in Tuberculosis Patients: A Meta-Analysis. Neuropsychiatr Dis Treat. 2022; 18:847-866. doi: 10.2147/NDT.S347579.
8. Mohammedhussein M, Hajure M, Shifa JE, Hassen TA. Perceived stigma among patient with pulmonary tuberculosis at public health facilities in southwest Ethiopia: A cross-sectional study. PLoS One. 2020;15(12):e0243433. doi: 10.1371/journal.pone.0243433.
9. DeSanto, D., Velen, K., Lessells, R. *et al.* A qualitative exploration into the presence of TB stigmatization across three districts in South Africa. *BMC Public Health* 2023; 23: 504. <https://doi.org/10.1186/s12889-023-15407->
10. Sweetland AC, Kritski A, Oquendo MA, et al. Addressing the tuberculosis-depression syndemic to end the tuberculosis epidemic. Int J Tuberc Lung Dis. 2017;21(8):852–61
11. DeVellis RF. Scale development: Theory and applications, 4^th^ edition, 2016.
12. Kroenke K, Spitzer RL, Williams JB. The Patient Health Questionnaire-2: Validity of a Two-Item Depression Screener. Medical Care. 2003;41:1284-92.
13. Kroenke K, Spitzer RL, Williams JB. 2001. The validity of a brief depression severity measure. *J Gen Intern Med* 2001; 16: 606-613.
14. Bhana A, Rathod SD, Selohilwe O, Kathree T, Petersen I. The validity of the Patient Health Questionnaire for screening depression in chronic care patients in primary care in South Africa. *BMC Psychiatry,* 2005;115: 118.
15. Van Rie A, Sengupta S, Pungrassami P, Balthip Q, Choonuan S, Kasetjaroen Y et al. Measuring stigma associated with tuberculosis and HIV/AIDS in southern Thailand: exploratory and confirmatory factor analyses of two new scales. Trop Med Int Health. 2008; 13(1):21-30. doi: 10.1111/j.1365-3156.2007.01971.x. PMID: 18290998.
16. Braun V, Clarke V. Reflecting on reflexive thematic analysis. Qualitative Research in Sport, Exercise and Health, 2019; 11(4), 589–597. https://doi.org/10.1080/2159676X.2019.1628806
